# A Thank You Note to Our Peer Reviewers (2025)

**DOI:** 10.1002/jia2.70205

**Published:** 2026-09-18

**Authors:** Kenneth H. Mayer, Annette H. Sohn, Linda‐Gail Bekker, Marlène Bras

**Affiliations:** ^1^ The Fenway Institute and Harvard Medical School Boston Massachusetts USA; ^2^ TREAT Asia, amfAR ‐ Foundation for AIDS Research Bangkok Thailand; ^3^ Desmond Tutu HIV Centre University of Cape Town Cape Town South Africa; ^4^ IAS – the International AIDS Society Geneva Switzerland

1

The *Journal of the International AIDS Society* (JIAS) would like to express our gratitude to the peer reviewers who contributed to reviewing articles for the journal in 2025, and to those senior scientists who have mentored early career researchers in reviewing.

Your time and expertise are crucial to upholding the quality of this publication, and we are thankful for your engagement.

We also wish to extend our appreciation to the JIAS Editorial Board members, Deputy Editors, Statistical Committee and Ethical Committee members for their valuable contributions in assessing and reviewing submitted articles.

Kenneth Mayer, co‐Editor‐in‐Chief

Annette Sohn, co‐Editor‐in‐Chief

Linda‐Gail Bekker, co‐Editor‐in‐Chief

Marlène Bras, Executive Editor

Aaloke Mody

Aditya Khanna

Adrien Allorant

Ahmad Haeri Mazanderani

Ajay Rangaraj

Akarin Hiransuthikul

Akel Azzi

Akshay Sharma

Alison Castle

Alison Hill

Alison Rodger

Allan Maleche

Amy Justice

Amy Zheng

Anchalee Avihingsanon

Andrainolo Ravalihasy

Andreas Haas

Andrew David Eaton

Andrew Gibbs

Andrew Grulich

Andrew Mujugira

Angela Bengtson

Ann Strode

Antoine Jaquet

Ariane van der Straten

Arman Oganisian

Ashley Underwood

Ashwin Belludi

Aster Gebremedhin

Audrey Pettifor

Bao Vu

Beatrice Wamuti

Benjamin Bavinton

Benjamin Brown

Benjamin Cheabu

Benjamin Chi

Beo Oliveira Leite

Bindiya Meggi

Brenda Crabtree‐Ramírez

Brett Millar

Brian Honermann

Brooke Nichols

Bruce Richman

Caitlin Dugdale

Caitlin Madevu‐Matson

Carina Marquez

Carol Strong

Catherine Godfrey

Catherine Martin

Cedric Bien‐Gund

Chelsea Coakley

Chenglin Hong

Chetna Jaiswal

Chisom Obiezu‐Umeh

Christina Lumbantoruan

Christopher Akolo

Clara Le Saux

Claudia Cortes

Cloete Van Vuuren

Colette Smith

Colleen Kelley

Corwin Coppinger

Curtis Chan

Dan Tran

Daniel Elakpa

Danielle Campbell

Darrell Tan

David Denning

David Kietrys

David Van de Vijver

Davide Moschese

Deanna Kerrigan

Debbie Humphries

Deepak Mattur

Deja Clement

Denis Nash

Denise Franzsen

Denise Jacobson

Diane Havlir

Diego Cecchini

Dion Allen

Dobromir Dimitrov

Dorlim Moiana Uetela

Dumbani Kayira

Eiichi N Kodama

Elaine Abrams

Elana Rosenthal

Elfriede Agyemang

Elijah Kakande

Elise Lankiewicz

Elizabeth Mayfield Arnold

Elizabeth Montgomery

Ellen Brazier

Elske Hoornenborg

Elzette Rousseau

Emily Hyle

Emily Namey

Emma Kalk

Erin Wilson

Esau Joao

Fabian Raeber

Fernanda Fonseca

Flavia Kiweewa

Gabriel Chamie

Gabriela Cromhout

Gabriela Patten

Gary Whitlock

Gede Benny Setia Wirawan

Gesine Meyer Rath

Grace Chung Yan Lui

Graham Taylor

Hae‐Young Kim

Harry Reyes Nieva

Heather‐Marie Schmidt

Helen Bygrave

Hermann Brou

Huub Gelderblom

Intira Collins

Isaac Núñez

Iuri C. Leite

Jacob Bleasdale

Jade Ghosn

Jake Pry

James Moody

Jason Johnson Peretz

Jason Reed

Jayne Lewis‐Kulzer

Jeanne Goupil De Bouille

Jeb Jones

Jef Vanhamel

Jennifer Belus

Jesse Heitner

Jessica Fogel

Jessica Haberer

Jillian Lau

Jillian Pintye

Jimmy Ma

Jirair Ratevosian

Joanne Stekler

Joep J. Van Oosterhout

John Humphrey

John Santelli

Jonathan Friedman

Jonathan Mwangi

Jonathan Ross

Joseph Cadelina

Joseph Larmarange

Joseph Rosen

Julia Roider

Julian Falutz

Julie Fox

Julie Somogyi

Junko Tanuma

Justen Manasa

Kai Jonas

Karine Dube

Karl Technau

Kasonde Mwaba

Katharine Bar

Kathleen MacQueen

Kathleen Powis

Katrina Ortblad

Kehinde Balogun

Kejal Hasmukharay

Kelika Konda

Kevin De Cock

Kevin MacKenzie

Kevin McKenzie

Kim Anderson

Kim Jonas

Kim Nguyen

Kim Steegen

Kimberly Powers

Kirsten Arendse

Lana Lee

Lara Vojnov

LaRon Nelson

Laure Stella Ghoma Linguissi

Lauren Cirrincione

Lauren Graybill

Lauren M. Hill

Laurent Mandelbrot

Lawrence Long

Lee Fairlie

Lee Sonia

Leigh Johnson

Leigh McClarty

Leslie Enane

Lindsey Reif

Linxuan Wu

Lisa Abuogi

Lisa Frigati

Lisa Jahn

Lucie Cluver

Maarten Reitsema

Mackenzie Cottrell

Mackenzie Stewart

Magdalene Walters

Maitreyi Sahu

Marcel Tongo

Margaret Mwangi

Mari Armstrong‐Hough

Maria Nardell

Maria Pyra

Marie‐Claude Lavoie

Marije Hoftra

Marina Caskey

Marina Klein

Marissa Becker

Marthe Le Prevost

Martin F Schim van der Loeff

Martin Holt

Mary Kathryn Grabowski

Matthew Hickey

Matthew Kusen

Matthew Spinelli

Maud Mavigner

Maureen Moyo Chilufya

Mayara Secco

Mhairi Maskew

Michael Christian

Michael Evangeli

Michael Jordan

Michael Martin

Michael Thomson

Mícheál Ó Breasail

Moses Bateganya

Mulualem Kelebie

Nagalingesawaran Kumarasamy

Nandita Sugandhi

Nanette Benbow

Natella Rakhmanina

Nathan A. Summers

Nathan Ford

Nel Jason Haw

Nicola Barnes

Nicola Jones

Nicolas Paul

Niklaus Daniel Labhardt

Nittaya Phanuphak

Noelle Le Tourneau

Nomcebo Mokgethi

Nora Rosenberg

Olivier Robineau

Olujuwon Ibiloye

Oluwaseyi Isehunwa

Otoyo Toyo

Pablo Rojo

Pat Flynn

Patricia Agaba

Peter Anderson

Peter Ghys

Peter Vickerman

Praphan Phanuphak

Pui Wong

Rachel Yorlets

Rami Kantor

Rebecca Zimba

Reena Rajasuriar

Rena Janamnuaysook

Rena Patel

Renee Heffron

Ricardo Baruch‐Dominguez

Richard Jefferys

Richard Lessells

Robert Peck

Robin Schaefer

Rodenie Olete

Rohit Ramaswamy

Ronnie Gravett

Rose Kaptchuk

Roy Gulick

Rutendo Bothma

Sabina Nduaguba

Sam Phiri

Sara Paparini

Sarah Charnaud

Sarah Christie

Sarah Kattakuzhy

Sarah Masyuko

Sarah Maylin

Sarah Roberts

Sarah Stansfield

Scholastic Ashaba

Sebastian Linnemayr

Seth Inzaule

Seul Ki Choi

Shashi Kapadia

Shashwatee Bagchi

Shawnalyn Sunagawa

Shuzo Matsushita

Simple Ouma

Siyanai Zhou

Stephen Asiimwe

Steven Deeks

Steven K. Grinspoon

Supanat Thitipatarakorn

Susan Buchbinder

Susanne Doblecki‐Lewis

Suzanne Leclerc‐Madlala

Suzanne McCluskey

Swarnali Goswami

Sylivia Nalubega

Tafadzwa Madanhire

Tamsin Phillips

Thayer Anderson

Theodore Ruel

Thomas Gachie

Tim Brown

Timothy R Broady

Tinashe Mudzviti

Tonia Poteat

Trevor Hart

Trudy Leong

Tsz Ho Kwan

Twaambo Euphemia Hamoonga

Tyson Arapali

Valentina Cambiano

Vamsi Vasireddy

Victor Pimentel

Virginia Fonner

Virginia Macdonald

Vita Jongen

Warittha Tieosapjaroen

Win Han

Xianming Zhu

Xin Niu

Yasmine Fadil

Yazdan Yazdanpanah

Zhixin Fan

Zhongdan Chen

